# Interpreting whole‐body carbohydrate oxidation and ‘oxidation efficiency’ in carbohydrate supplement studies

**DOI:** 10.1113/EP093566

**Published:** 2026-01-20

**Authors:** Stefan Pettersson

**Affiliations:** ^1^ Center for Health and Performance, Department of Food and Nutrition, and Sport Science University of Gothenburg Gothenburg Sweden

1

I read with interest the article by Dean et al. (2026), ‘Comparative effects of a glucose–fructose bar, glucose–fructose hydrogel and maltodextrin gel on carbohydrate oxidation and sprint performance in Tier 2 athletes’ in *Experimental Physiology*. Independent comparisons of commercial carbohydrate supplements are valuable for practitioners and athletes. I would, however, like to raise several methodological and interpretative points that constrain the strength of the inferences that can be drawn from the reported data.

First, whole‐body indirect calorimetry in an oral glucose tolerance test (OGTT) cannot resolve exogenous from endogenous carbohydrate oxidation. In the Introduction, the modified OGTT is described as being performed ‘to … determine differences in substrate metabolism between products’. Indirect calorimetry quantifies total carbohydrate oxidation from all sources combined; it cannot separate oxidation of the 45 g of ingested carbohydrate from ongoing hepatic glucose production and muscle glycogen use (Frayn, 1983; Jeukendrup & Wallis, 2005). Thus, between‐trial differences in carbohydrate oxidation reflect overall whole‐body metabolic responses rather than product‐specific exogenous carbohydrate metabolism.

This limitation is acknowledged in the ‘Limitations’ section (‘without tracers, it is not possible to distinguish between exogenous and endogenous oxidation’), but elsewhere in the manuscript differences in carbohydrate oxidation and ‘oxidation efficiency’ are sometimes discussed as if they directly reflect oxidation of the ingested products.

Second, ‘oxidation efficiency’ cannot be interpreted as the percentage of ingested carbohydrate oxidized. ‘Carbohydrate oxidation efficiency’ is defined as (total carbohydrate oxidized/45 g) × 100 and interpreted as ‘the percentage of the ingested carbohydrate (45 g) that was oxidized’, with the statement that this ‘provides a good estimation in the absence of ^13^C tracer data (Hulston et al., 2009)’. However, the Frayn equation used (1983) provides only total whole‐body carbohydrate oxidation. Without labelled substrates, this index cannot distinguish exogenous from endogenous sources and therefore cannot be interpreted literally as the proportion of ingested carbohydrate that was oxidized.

Hulston et al. (2009) instead illustrate that tracer methodology is required to separate exogenous from endogenous oxidation: they used ^13^C‐labelled substrates for precisely this purpose. The individual data in their figure 3b further highlight the limitation of the index. Values approaching 100% would imply that the amount of carbohydrate oxidized at rest over 60 min equalled the entire 45 g provided by the supplements, which, at such doses and in resting conditions, would necessarily include substantial endogenous carbohydrate use. The large within‐subject variation in ‘efficiency’ between the three products (differences of 40–70 percentage points in some individuals) is also more plausibly explained by day‐to‐day variation in basal metabolism and endogenous substrate use, with Dean et al.’s instruction to “continue their typical diet in the days leading up to each visit” allowing variation in baseline substrate oxidation related to prior dietary macronutrient composition (Miles‐Chan et al., 2015), than by large differences in true exogenous oxidation among products composed of rapidly absorbed carbohydrates; within‐subject variability is also evident in repeated 60‐min resting measures even under highly controlled respiration‐chamber conditions (Mähler et al., 2023). Moreover, because the ingested dose was identical across conditions, ‘oxidation efficiency’ is simply total carbohydrate oxidation normalised to the ingested dose; thus, Figure 3b is a linear rescaling of Figure 3a and should not be treated as an independent outcome or interpreted as exogenous carbohydrate oxidation.

Third, the interpretation of time‐course data in the absence of a significant product × time interaction is questionable. For carbohydrate oxidation per minute, the two‐way repeated‐measures ANOVA showed significant main effects of product and time but no significant product × time interaction (*P* = 0.201). In this context, *post hoc* comparisons at individual time points (15, 40 and 50 min, emphasized in figure 4a Dean et al. 2026) are best viewed as exploratory rather than as evidence for time‐specific product effects. It would be helpful if the manuscript more clearly distinguished between overall (time‐averaged) product differences and exploratory analyses at individual time points.

Fourth, the mechanistic interpretation of small differences in resting fat oxidation and their relationship to performance is questionable. Over the 60 min OGTT, total fat oxidation differed by ∼2 g between GF‐Bar (7.4 g) and MD‐Gel (9.5 g), whereas mean respiratory quotient values were very similar (0.86 and 0.83). This difference of ∼2 g of fat, equivalent to ∼18 kcal (Peronnet & Massicotte, 1991), is small in absolute terms during 1 h of rest, yet the Discussion interprets this as a greater ‘suppression of fat oxidation’ in GF‐Bar, ‘indicative of increased carbohydrate availability’, invokes Randle cycle mechanisms and suggests implications for ‘ergogenic potential’.

Given that these measurements reflect whole‐body substrate use at rest, with modest absolute differences and limited duration of the protocol, such mechanistic inferences seem stronger than the data support, especially given that no ergogenic effect was seen in the subsequent sprint trial (all *P* > 0.05). Moreover, only 5 of the 16 participants from the OGTT also took part in the exercise trial, limiting the extent to which resting metabolic responses can be linked to performance outcomes within individuals. The Discussion itself notes that ‘no one product was superior in maintaining performance’ and that carbohydrate effects ‘may be more relevant to prolonged or glycogen‐depleting exercise’, underscoring this disconnect.

In summary, the reliance on whole‐body indirect calorimetry with limited standardization and without tracer methods, the definition and interpretation of ‘oxidation efficiency’, the emphasis placed on *post hoc* time‐point differences in the absence of a significant interaction, and the mechanistic extrapolation from small differences in resting fat oxidation despite partial overlap between OGTT and exercise samples and no observed performance effects all limit the strength of conclusions regarding product‐specific metabolic superiority.

These points do not detract from the value of comparing commonly used commercial supplements, but they do suggest that the conclusions should remain tightly aligned with what the methods can support. Future studies of carbohydrate supplements would benefit from incorporating stable‐isotope tracers to separate exogenous from endogenous substrate oxidation and from using protocols matched to the mechanistic and/or performance questions posed.
